# Cervical Epidural Electrical Stimulation Increases Respiratory Activity through Somatostatin-Expressing Neurons in the Dorsal Cervical Spinal Cord in Rats

**DOI:** 10.1523/JNEUROSCI.1958-21.2022

**Published:** 2023-01-18

**Authors:** Erika L. Galer, Ruyi Huang, Meghna Madhavan, Emily Wang, Yan Zhou, James C. Leiter, Daniel C. Lu

**Affiliations:** ^1^Department of Neurosurgery, University of California Los Angeles, Los Angeles 90095, California; ^2^Department of Molecular Cellular and Integrative Physiology, University of California Los Angeles, Los Angeles 90095, California; ^3^Brain Research Institute, University of California Los Angeles, Los Angeles 90095, California; ^4^Research Service, White River Junction VA Medical Center, White River Junction 05009, Vermont

**Keywords:** cervical spinal cord, epidural stimulation, somatostatin

## Abstract

We tested the hypothesis that dorsal cervical epidural electrical stimulation (CEES) increases respiratory activity in male and female anesthetized rats. Respiratory frequency and minute ventilation were significantly increased when CEES was applied dorsally to the C2–C6 region of the cervical spinal cord. By injecting pseudorabies virus into the diaphragm and using c-Fos activity to identify neurons activated during CEES, we found neurons in the dorsal horn of the cervical spinal cord in which c-Fos and pseudorabies were co-localized, and these neurons expressed somatostatin (SST). Using dual viral infection to express the inhibitory Designer Receptors Exclusively Activated by Designer Drugs (DREADD), hM4D(Gi), selectively in SST-positive cells, we inhibited SST-expressing neurons by administering Clozapine N-oxide (CNO). During CNO-mediated inhibition of SST-expressing cervical spinal neurons, the respiratory excitation elicited by CEES was diminished. Thus, dorsal cervical epidural stimulation activated SST-expressing neurons in the cervical spinal cord, likely interneurons, that communicated with the respiratory pattern generating network to effect changes in ventilation.

**SIGNIFICANCE STATEMENT** A network of pontomedullary neurons within the brainstem generates respiratory behaviors that are susceptible to modulation by a variety of inputs; spinal sensory and motor circuits modulate and adapt this output to meet the demands placed on the respiratory system. We explored dorsal cervical epidural electrical stimulation (CEES) excitation of spinal circuits to increase ventilation in rats. We identified dorsal somatostatin (SST)-expressing neurons in the cervical spinal cord that were activated (c-Fos-positive) by CEES. CEES no longer stimulated ventilation during inhibition of SST-expressing spinal neuronal activity, thereby demonstrating that spinal SST neurons participate in the activation of respiratory circuits affected by CEES. This work establishes a mechanistic foundation to repurpose a clinically accessible neuromodulatory therapy to activate respiratory circuits and stimulate ventilation.

## Introduction

Breathing is essential to provide adequate O_2_/CO_2_ exchange in mammals and contributes to many homeostatic, nonrespiratory behaviors (Bartlett and Leiter, 2012). Given the central role of respiration in numerous homeostatic processes, compromised respiratory function leads to significant morbidity and mortality. The primary aid to respiratory deficiencies is mechanical ventilation, which is an imperfect therapy for respiratory insufficiency and is strongly associated with critical illness, and extended use of mechanical ventilation increases the risk of death (Combes et al., 2003; Loss et al., 2015). Therefore, innovative therapeutic approaches to enhance respiratory activity may forestall the need for mechanical ventilation and may prove beneficial in various cardiorespiratory disorders.

The rhythm and the pattern of respiratory activity originate in a network of pontomedullary neurons that rhythmically activate phrenic and other respiratory-related motor neurons in the pons, medulla, and spinal cord that, in turn, innervate the respiratory muscles, especially the diaphragm (Dobbins and Feldman, 1994; Feldman and Del Negro, 2006; Smith et al., 2013; Cui et al., 2016). The internal organization of the pontomedullary respiratory control system is complex, serves multiple overlapping functions, and is challenging to access in humans. These factors limit therapeutic interventions in the brainstem to modulate respiratory function. The spinal cord may provide an alternate, less invasive route through which the neural respiratory circuitry may be accessed. Spinal respiratory interneurons, firing before, during, and after phrenic nerve activity, modulate respiratory activity (Bellingham and Lipski, 1990; Cleland and Getting, 1993; Oku et al., 2008; Ghali, 2018; Jensen et al., 2020). Additionally, spinal interneurons and propriospinal neurons can initiate phrenic motor neuron activity in the absence of supraspinal input, usually when certain restricted conditions are met (i.e., reduced preparations, inhibition of fast inhibitory activity and activation of glutamatergic excitatory neurons; Coglianese et al., 1977; Viala et al., 1979; Ghali and Marchenko, 2016; Le Gal et al., 2016; Cregg et al., 2017). Thus, the spinal cord is a mediator of ascending respiratory sensory input and descending respiratory motor output and may contain auxiliary respiratory circuits that subserve the activity of the pontomedullary respiratory controller that can, in special circumstances (reduced experimental models in animals), provide an endogenous respiratory drive. Cervical epidural electrical stimulation (CEES) increases respiratory activity in anesthetized mice and anesthetized humans (Huang et al., 2016, 2022). Yet, the cells and mechanisms responsible for the respiratory activity elicited by CEES remain unknown. Therefore, we sought to identify the neuronal cell type(s) mediating the respiratory responses to CEES in anesthetized rats.

Consistent with previous observation in mice (Huang et al., 2016), respiratory activity, frequency and tidal volume, increased when CEES was applied at cervical levels C2/3–C6 in anesthetized rats. After demonstrating that CEES increased respiratory activity, we sought to identify the cell type(s) that may be involved in the modulation of respiration by CEES. We identified putative spinal respiratory interneurons using the retrograde transsynaptic tracer pseudorabies virus (PRV-152) and then used expression of the immediate early gene, c-Fos, following CEES to identify spinal neurons mediating the increase in respiratory activity by CEES. Somatostatin (SST) is expressed in some of the medullary elements of the respiratory central pattern generator (Stornetta et al., 2003; Tan et al., 2008), and we hypothesized that somatostatin might also be expressed in the spinal circuit that is responsible for modulation of respiratory activity induced by CEES. Using two adeno-associated viral (AAV) vectors, we selectively expressed a double-floxed inhibitory Designer Receptors Exclusively Activated by Designer Drugs (DREADD), hM4D(Gi), in SST-expressing cervical spinal neurons and tested the hypothesis that inhibition of these cervical, dorsal, spinal neurons would block the respiratory response to CEES. The results of this sequence of studies indicate that SST-positive cervical neurons are associated anatomically with CEES-elicited respiratory activity, as indicated by co-localization of SST, c-Fos elicited by CEES, and pseudorabies virus injected into the diaphragm, which defines the intersection of neurons activated by CEES and neurons associated with activation of the diaphragm. Thus, SST-positive neurons in the cervical spinal cord may play a key role in mediating the respiratory response to CEES.

## Materials and Methods

Mixed-gender Sprague Dawley rats (250–350 g, *n* = 49) were purchased from Envigo and allowed to acclimate in the University of California Los Angeles vivarium for one week. Animals were kept in 12/12 h light/dark cycle with *ad libitum* access to standard food and water. All procedures were approved by the University of California Animal Research Committee (protocol #2014-122) and were conducted in accordance with the *Guide for the Care and Use of Laboratory Animals* of the National Institutes of Health.

### Pseudorabies virus polysynaptic retrograde tracing

Animals (*n* = 12) were anesthetized with isoflurane and a horizontal abdominal incision was performed to expose the diaphragm. The Bartha strain of pseudorabies virus (PRV-152), supplied by the Center for Neuroanatomy with Neurotropic Viruses, NIH Virus Center P40 OD010996, was injected into the diaphragm of animals to label spinal respiratory-related neurons. Four 10-μl injections of 9 × 10^8^ pfu/ml of PRV-152 were made bilaterally into the diaphragm using a Hamilton syringe and 30-ga needle (Lane et al., 2008). The abdominal tissue was closed with 5-0 Vicryl, and the skin incision was closed with staples. Animals were housed in a biohazard vivarium for 60 h, after which they were transported to the lab and prepped for CEES experiments.

### Epidural electrical stimulation at C2/3 in PRV-152-expressing rats and c-Fos activation

The CEES studies were conducted 64–66 h after PRV injections in each animal to maximize polysynaptic transport and minimize immune cell infiltration. It is possible that some of the cells expressing PRV-GFP were glia, as these cells phagocytize the debris from infected and lysed cells. However, glia are unlikely to fluoresce with GFP since we used a less virulent PRV and relatively short incubation times (Rinaman et al., 1993). Labeling glia through synaptic transfer is unlikely as this has not been observed in glial cells (Rinaman et al., 1993). The interval between PRV injection and CEES allowed sufficient time for polysynaptic labeling of premotor neurons and spinal respiratory-related interneurons (Dobbins and Feldman, 1994; Lane et al., 2008). During each CEES experiment, the rat was kept on a water-circulating heating pad to prevent hypothermia. Animals were anesthetized with urethane (1200 mg/kg) and α-chloralose (30 mg/kg). A vertical incision was made ventrally on the neck; the sternohyoid muscles were separated to expose the trachea; a small incision was made between the cartilage rings of the trachea; a short segment of PE 200 tubing was inserted to tracheostomize each animal; and the tracheostomy tube was connected to a pneumotach (Validyne) to record respiratory airflow. Two wires (St. Steel 7 Strand, AM-Systems), with the insulation stripped at the end (2 mm), were inserted bilaterally through abdominal incisions into the lateral costal portion of the diaphragm muscle to record electromyographic (EMG) activity. Each animal was placed prone and a laminectomy was performed to expose C2–C7 spinal cord levels. Dorsal CEES was administered using a stimulating electrode (Tungsten Parylene 0.01, AM-Systems) placed ∼2 mm lateral to the midline on the dorsal surface of the cervical spinal cord, and a ground electrode was placed on the dorsal surface of the spinal cord ∼2–3 mm away from the stimulating electrode. The ends of the stimulating and ground electrodes were stripped, leaving ∼2 mm of each electrode tip uninsulated. CEES was delivered as a continuous 30-Hz monophasic (500-μs pulse width) train of impulses for 30 s (Master 9 AMPI). EMG signals were amplified 1000× and bandpass filtered at 300–1000 Hz before digitization. Diaphragmatic activity was sampled throughout each study at a rate of 2 kHz.

Animals were randomized to receive six trials of 30-s active CEES (*n* = 9) and sham stimulation or sham (*n* = 3) stimulation only at the intersection of cervical levels 2 and 3 (C2/3). Sham stimulation trials in the CEES group were performed to control for any effects that the electrode pressure on the dura may have had on respiratory behavior in the absence of current. Experiments in which animals received only sham stimulation were performed as a control for c-Fos expression in the unstimulated condition. To execute the sham trials, the stimulation and ground electrodes were placed on the dura with similar pressure as stimulation trials, but no stimulation was delivered. During sham stimulation (Sham) and active stimulation trials (Stim), data were recorded for 1 min of baseline recording (Pre), 30 s. Stim/Sham, and 8–10 min post-Stim/Sham. Data presented are the 30 s before Stim/Sham (Pre), 30 s of Stim/Sham (Intra), and 30 s of after Stim/Sham (Post). Each animal was allowed to survive for at least 1 h after the mid-way point of stimulation to allow c-Fos expression to develop.

### Visualization of PRV-152, c-Fos, and somatostatin

To identify candidate neurons mediating the respiratory effects of CEES, we studied co-localization of PRV-152 (putative respiratory interneurons) and c-Fos (an immediate early gene likely activated by CEES) and co-localization of somatostatin (also a possible marker of interneurons mediating the effects of CEES in the cervical spinal cord). To conduct these studies, animals were perfused transcardially with PBS followed by 4% paraformaldehyde (pH 7.3) at the end of each experiment. Tissue was extracted and postfixed for 24 h in 4% paraformaldehyde, placed in 30% sucrose for cryopreservation, and placed into 10% gelatin and frozen. Tissue was sectioned into 30-μm coronal slices using a cryostat (Leica CM 1800). PRV-152 encodes a GFP tag for localization that was amplified using an antibody against the GFP protein. Tissue was incubated in primary antibodies for 48 h [anti-c-Fos (1:1000), Abcam ab190289; anti-green fluorescent protein (GFP; 1:2500), Abcam, ab13970, mouse anti-somatostatin (1:50), Genetex, GTX71935]. Tissue was subsequently incubated in secondary antibodies against the host of the primary antibody for 1 h at room temperature (Cy3, Cy2, and Cy5; Jackson ImmunoResearch). Negative controls, in which tissue was subjected to the same protocol, but the primary antibody was not included, were used to confirm optimal antibody dilution and imaging settings. An additional negative control experiment was performed to ensure that the SST antibody was specific to the SST protein. In this experiment, an SST blocking peptide (MyBioSource) was preincubated (5 μg/ml) for 1 h at room temperature followed by a 24-h incubation with the primary antibody (ratio 10:1 of blocking peptide to primary antibody) at 4°C. The secondary antibody incubation was performed as described above. ImageJ was used to quantify cell expression and co-localization. Images were down sampled to an eight-bit resolution to facilitate cell counting, and the Threshold and Analyze Particle tools were used to quantify cells expressing c-Fos, GFP, and DAPI. GFP and c-Fos images were acquired and quantified at 10× magnification. Co-localization was quantified by merging the two expression images within ImageJ. Co-localization was considered positive when expression distributions within a cell were overlapping. The dorsal motor nucleus (DMN), which innervates organs related to the gastrointestinal tract, was explored in each animal to verify that PRV-152 did not leak into the abdominal space where it might have led to nonspecific spinal labeling.

### C3–C6 cervical epidural electrical stimulation induced respiratory activity

Seven animals underwent CEES at multiple cervical levels to map the respiratory responses to EES delivered along the cervical spinal cord (C3, C4, C5, and C6). Three animals underwent CEES at a constant location (C2/3) to determine the respiratory responses at different stimulation amplitudes between 0.5 and 3 mA (data not shown). Each animal was anesthetized and prepared as described above, but these animals did not receive PRV-152 injections into the diaphragm before the CEES studies.

### Inhibitory DREADD expression in somatostatin-expressing neurons in the dorsal cervical spinal cord

Two viral constructs were injected to achieve selective expression of the inhibitory DREADD, HM4D(Gi), in somatostatin (SST)-expressing neurons. Dual AAV intraspinal injections were performed in 27 animals (*n* = 27). Rats that received both viral constructs, SST-Cre and hM4D(Gi), were divided into a CNO-test group (AAV-SST-Cre+AAV-hM4D(Gi)+CNO in dimethylsulfoxide (DMSO), *n* = 9 of 27) and a vehicle control group that received DMSO alone (AAV-SST-Cre+AAV-HM4D(Gi)+DMSO, *n* = 9 of 27). Additionally, nine animals were injected with an AAV lacking the Cre construct (AAV-SST-eGFP) and the AAV- hM4D(Gi)-mCherry to serve as a viral expression control (these animals would not be expected to express the inhibitory hM4D(Gi) or mCherry). These animals received CNO and CEES as described below.

Each animal in the DREADD studies was anesthetized with isoflurane and placed prone on a water-circulating heating pad to prevent hypothermia during the injection of AAV constructs. Surgery was performed aseptically: the skin was shaved and prepped with 70% alcohol and povidone/iodine. A vertical incision was made from the base of the skull to the top of the shoulder blades to gain access to the dorsal spinal cord. The acromiotrapezius and paraspinal muscles were separated to expose the spinal column. A laminectomy at cervical level 3 (C3) was performed. Four intraspinal microinjections of AAV serotype 2 (AAV2) carrying Cre under control of the SST promoter and expressing enhanced green fluorescent protein (eGFP): AAV2-SST-eGFP-T2A-iCre-WPRE (Vector Biolabs; 3.0 × 10^12^ gc/ml) were made. An additional AAV carrying a double-floxed inhibitory DREADD receptor protein, hM4D(Gi), fused to the mCherry protein (mCherry; Krashes et al., 2011): AAV-hSyn-DIO-hM4D(Gi)-mCherry (Addgene; 2.5 × 10^12^ gc/ml) was mixed with the other AAV virus, and the injections were performed (*n* = 18 of 27 rats). To control for any effect that expression of the viral vector or CNO may have had, nine of the 27 animals received intraspinal injections of a control adenoviral vector that did not include the Cre cassette, AAV2-SST-eGFP-WPRE (Vector Biolabs, 3.0 × 10^12^ gc/ml), but these animals still received the same double-floxed AAV-hSyn-DIO-hM4D(Gi)-mCherry (Addgene; 2.5 × 10^12^ gc/ml). Injections were targeted rostrally to C2/3 and caudally to C3/4. Injections were made bilaterally ∼1 mm lateral from the posterior central vein. All injections were performed at 2 nl/s at a depth of 0.5–1.0 mm from the dorsal surface using a micropressure injector (WPI, Micro2T; Mondello et al., 2018). A 5-min period elapsed before the needle was withdrawn from the tissue to minimize leakage. Each animal received buprenorphine (0.05 mg/kg) before closing the surgical incision. The muscles were closed with 5-0 Vicryl, and 5-0 Ethilon was used to close the skin tissue. Carprofen (5 mg/kg) was administered, as needed for dermatitis, when it developed around the incision site.

### Effect of epidural electrical stimulation during inhibition of somatostatin-expressing neurons

Three weeks elapsed between the time of AAV-hM4D(Gi) plus AAV-SST-Cre-eGFP injections, and the study of respiratory activity during CEES with or without activation of the inhibitory DREAAD channel. For each CEES study, the animal was anesthetized with urethane (1200 mg/kg) and α-chloralose (30 mg/kg), and prepared with diaphragm EMG electrodes, a tracheostomy, and a laminectomy, as described above. Animals underwent sham and active CEES at C3 to define the stimulation amplitude to use and the typical respiratory response of each animal before drug or vehicle injection. Amplitudes were selected to achieve respiratory responses without overt upper extremity muscle activity and ranged from 1.0 to 2.5 mA. After successful respiratory modulation by CEES, each animal was given 1 mg/kg CNO (in 1.5% DMSO) intraperitoneally to activate the hM4D(Gi) or a control injection of 1.5% DMSO to assess the effects of the vehicle. High doses of CNO may affect baseline behavior without the expression of a DREADD receptor (MacLaren et al., 2016; Goutaudier et al., 2019; Martinez et al., 2019). To mitigate the likelihood of such effects, we used a low-dose of CNO (MacLaren et al., 2016), and we included a control group of animals (AAV-SST-eGFP+AAV-hM4D(Gi)+CNO) that received viral constructs that did not result in the expression of the hM4D(Gi) receptor, and injections of CNO. Animals underwent sham trials of CEES before and 20 and 60 min post-drug delivery. Active stimulation trials were conducted every 20 min for 100 min following drug delivery. Animals that had minimal EES respiratory modulation before drug delivery were excluded from the data analysis (*n* = 3 of 27).

### Quantification of hM4Dgi-mCherry expression

At the end of the experiment, the animals were perfused transcardially, and tissue was prepared for immunohistochemical studies as described above. Thirty μm coronal slices from the cervical spinal cord were incubated in primary antibodies for 24 h at 4°C, anti-mCherry (1:200) GeneTex, GTX128508; rabbit anti-SST (1:50) Bioss, 8877R. Incubation in secondary antibodies (1:200) was performed for 1 h at room temperature. Images with 16-bit resolution were acquired using a digital microscope (Echo Revolve, Echo). ImageJ was used to quantify cell expression and assess co-localization as described above. Images labeled with DAPI and mCherry were acquired and quantified at 20× magnification.

### Data analysis

In the first study using PVR-152 and c-Fos activation, respiratory and EMG data were obtained with DataView (W. J. Heitler, University of St. Andrews). End-tidal CO_2_ (Petco_2_) values were obtained using a Kent Scientific (capnograph and recorded using LabChart, ADInstruments). Data visualization, postprocessing, and extraction were performed with MATLAB (MathWorks). Tidal volumes were calculated from the integral of the inspiratory flow, and minute ventilation was calculated as the product of the respiratory frequency multiplied by the tidal volume. Diaphragm activity during C2/3 CEES was calculated as the average integral of diaphragm EMG activity and expressed as a percent of the difference from baseline activity. The data were tested for normality using the Shapiro–Wilkes test for normality and normality was verified. Statistics were performed in R Studio. C2/3 EES respiratory frequency, minute ventilation, and Petco_2_ were analyzed using a two-way repeated measures ANOVA in which treatment condition versus sham and time (serial measurements) were within subjects factors. Tukey's honestly significant test was performed for *post hoc* analysis when the results of the ANOVA indicated that paired comparisons were permissible. To analyze c-Fos and PRV-152 expression, we used a two-way ANOVA with Sham/CEES as a between-subject factor and cervical level (C1–C7) as a within subject factor.

In the study mapping the responses to CEES, the respiratory frequency, minute ventilation, and Petco_2_ during CEES at C3–C6 were analyzed with discrete two-way ANOVAs for each location tested (C3–C6). The data were tested for normality using the Shapiro–Wilkes test for normality and normality of the data distribution was confirmed. Treatment (Sham or Stim) and time were within subject factors. To explore if any one location initiated an increase in respiratory activity more than another, a three-way ANOVA was used (treatment condition by time by cervical location).

To analyze the results of DREADD activation during CEES, we analyzed respiratory activity as a percent change calculated as the frequency or minute ventilation change during the 30 s of Sham/Stim (Intra) compared with the frequency or minute ventilation of the 30 s immediately prior (Pre) divided by the 30 s immediately prior (Pre) multiplied by 100. The data, percentage change in frequency or minute ventilation, were tested for normality using the Shapiro–Wilkes test for normality and it was necessary to log transform the MV data before analysis. The transformed data were analyzed using a mixed effects model with treatment group (CNO or vehicle) as a between subjects factor and time as a within subjects factor. Multiple comparisons were made to compare each group to its own Sham condition and tested with Dunnett's test when the mixed effects model indicated that paired tests were warranted.

## Results

### Dorsal epidural electrical stimulation at C2/3 increases ventilation

**Figure 1.**
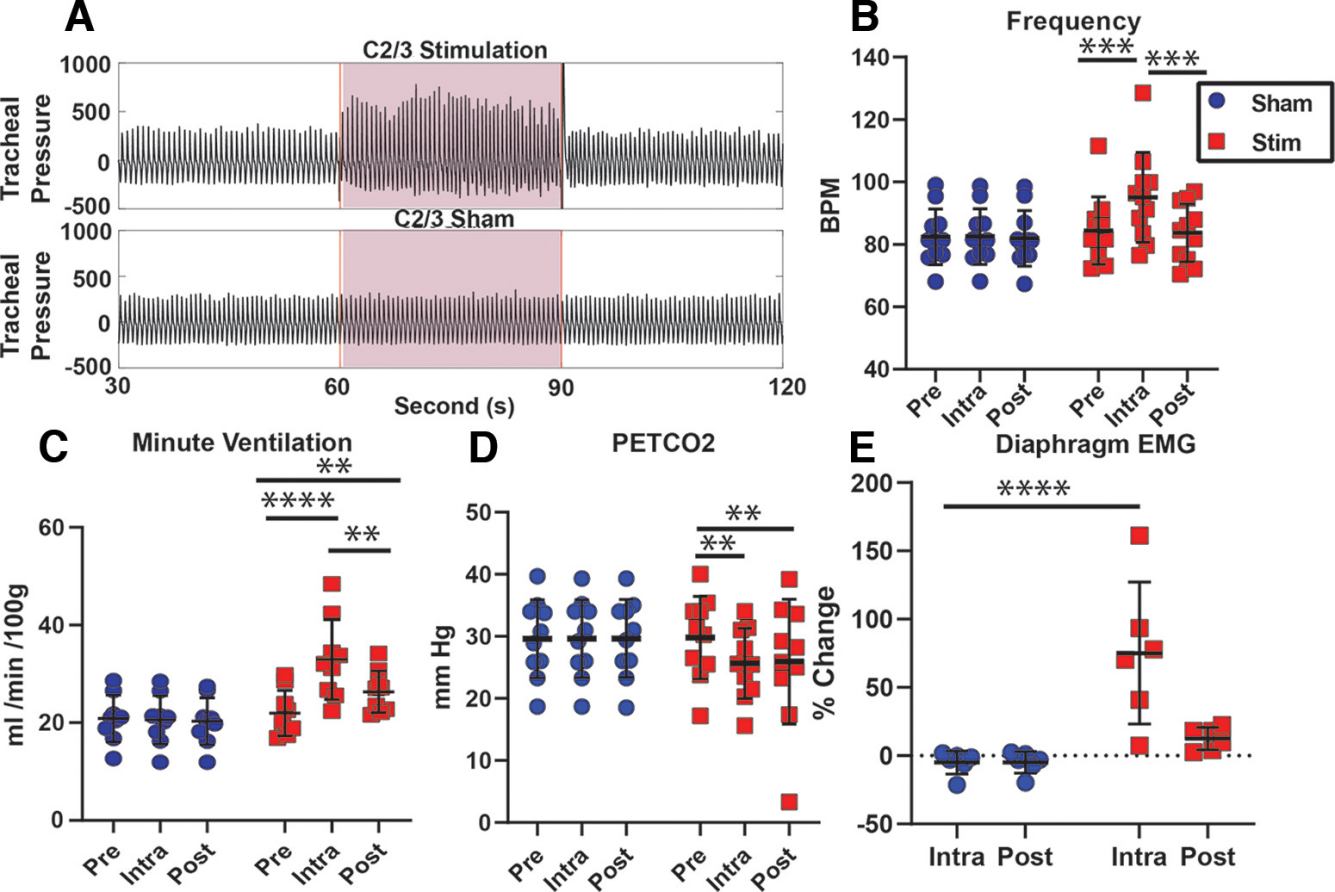
Cervical epidural electrical stimulation at the intersection of C2 and C3 increases respiratory activity in anesthetized rats. ***A***, Example of tracheal pressure recording from one animal receiving both Stimulation and Sham stimulation. ***B***, CEES increased respiratory frequency compared with baseline (Pre). Sham stimulation had no effect on respiratory frequency (*n* = 11). ***C***, CEES at C2/3 increased respiratory minute ventilation compared with baseline and was significantly greater in the Post period. Sham stimulation had no effect on minute ventilation (*n* = 10). ***D***, Petco_2_ was significantly decreased during and for 30 s after CEES (Post) compared with baseline (*n* = 10). ***E***, Diaphragm muscle activity as a percentage of the activity from baseline was increased during stimulation compared with Sham conditions (*n* = 6). Data were analyzed with a two-way ANOVA and Tukey's honestly significant test was used to adjust for multiple comparisons; **p < 0.01, ***p < 0.001, ****p < 0.0001. Mean line and error bars represent standard deviation.

To investigate respiratory responses to cervical spinal stimulation in anesthetized rats, CEES was applied to the dorsal epidural surface of the spinal cord at C2/3 using a continuous 30 Hz monophasic waveform for 30 s with amplitudes ranging from 1.0 to 2.5 mA (Fig. 1). There was a significant interaction between condition (Stim/Sham) and time for the respiratory frequency (*F*_(2,20)_ = 12.15, df = 2, *p* = 0.0004). Thus, the respiratory frequency changed significantly between baseline and the stimulated condition: CEES at C2/3 significantly increased the respiratory rate during stimulation compared with the baseline period (*p* = 0.0001; Table 1; Fig. 1*B*). Sham trials, in which the electrodes were placed on the epidural surface, but no current was delivered, had no effect on the respiratory rate compared with baseline (*p* > 0.05; Table 1; Fig. 1*B*). There was a significant interaction between condition and time for minute ventilation (*F*_(2,16)_ = 21.73, df = 2, *p* = 0.0001). Minute volume was significantly increased during CEES trials at C2/3 compared with baseline (*p* = 0.0001; Table 1; Fig. 1*C*). There was no effect on minute ventilation during sham trials compared with baseline (*p* > 0.05). Heart rate was assessed where possible to explore the cardiorespiratory effects C2/3 stimulation. Stimulation artifact often obscured the EKG signal, however, an average increase of 16 beats per minute (a 5–7% change in heart rate relative to the prestimulation values) was observed after the stimulation ended (data not shown). This increase was not observed after sham trials.

**Table 1.** Mean and SD of respiratory frequency, minute ventilation, the integrated EMG, and Petco_2_ before, during, and after CEES or sham treatment

Variable	Baseline (Pre); mean ± SD	During stimulation (Intra); mean ± SD	After stimulation (Post); mean ± SD
Condition: CEES			
Respiratory frequency (breaths/min)	84.4 ± 10.8	95.1 ± 14.4****	83.7 ± 9.3*^n.s.^*
Minute ventilation (ml/min/100 g)	21.99 ± 4.66	32.99 ± 8.21****	26.34 ± 4.26 **
Integrated diaphragmatic EMG (percent change)	0 ± 0	75.2 ± 52.0*	12.5 ± 8.27*^n.s.^*
Petco_2_ (mmHg)	29.80 ± 6.65	25.63 ± 5.67**	25.93 ± 10.07**
Condition: sham			
Respiratory frequency (breaths/min)	82.4 ± 8.9	82.5 ± 8.9	81.9 ± 8.9
Minute ventilation (ml/min/100 g)	20.99 ± 4.76	20.61 ± 4.76	20.33 ± 4.80
Integrated diaphragmatic EMG (percent change)	0 ± 0	−4.9 ± 8.4	−4.9 ± 7.9
Petco_2_ (mmHg)	29.64 ± 6.27	29.56 ± 6.29	29.66 ± 6.26

*p ≤ 0.05,

**p ≤ 0.01,

***p ≤ 0.001,

****p ≤ 0.0001; *n.s.*, not significant.

Diaphragm muscle activity and Petco_2_ were monitored to assess the ventilatory effect of CEES, as shown in Figure 1*D*,*E*. A significant interaction between condition and time was observed for Petco_2_ (*F*_(2,36)_ = 23.7, df = 2, *p* < 0.0001). Petco_2_ was significantly decreased during CEES compared with baseline (*p* = 0.002; Fig. 1*D*). Sham trials had no effect on Petco_2_ values. Because of stimulation artifact, diaphragm activity could only be identified in six animals during stimulation. Nonetheless, there was a significant interaction between condition and time for the diaphragm activity, expressed as a percent of the difference between the activity that was calculated for the Intra and Pre periods (*F*_(1,10)_ = 9.24, df = 1, *p* = 0.01). CEES significantly increased diaphragm activity compared with sham trials (Fig. 1*E*).

While respiratory activity was increased during stimulation, the changes in frequency and diaphragm activity did not last after stimulation ended, and all variables returned to near baseline levels after CEES ceased (*p* > 0.05; Fig. 1*B*,*E*). Minute ventilation remained significantly elevated compared with baseline levels (*p* = 0.006; Fig. 1*C*), and Petco_2_ remained significantly decreased post-stimulation (*p* = 0.001; Fig. 1*D*), reflecting the slower dynamics of CO_2_ accumulation in the blood. These results indicate that, similar to mice and humans, dorsal CEES increases respiratory activity in rats, and some, but not all, respiratory effects persisted briefly after the stimulation ended. However, all variables tended to decay back to baseline over ∼90 s (Huang et al., 2016, 2022).

### Epidural electrical stimulation activated cervical spinal sensory and respiratory interneurons

**Figure 2.**
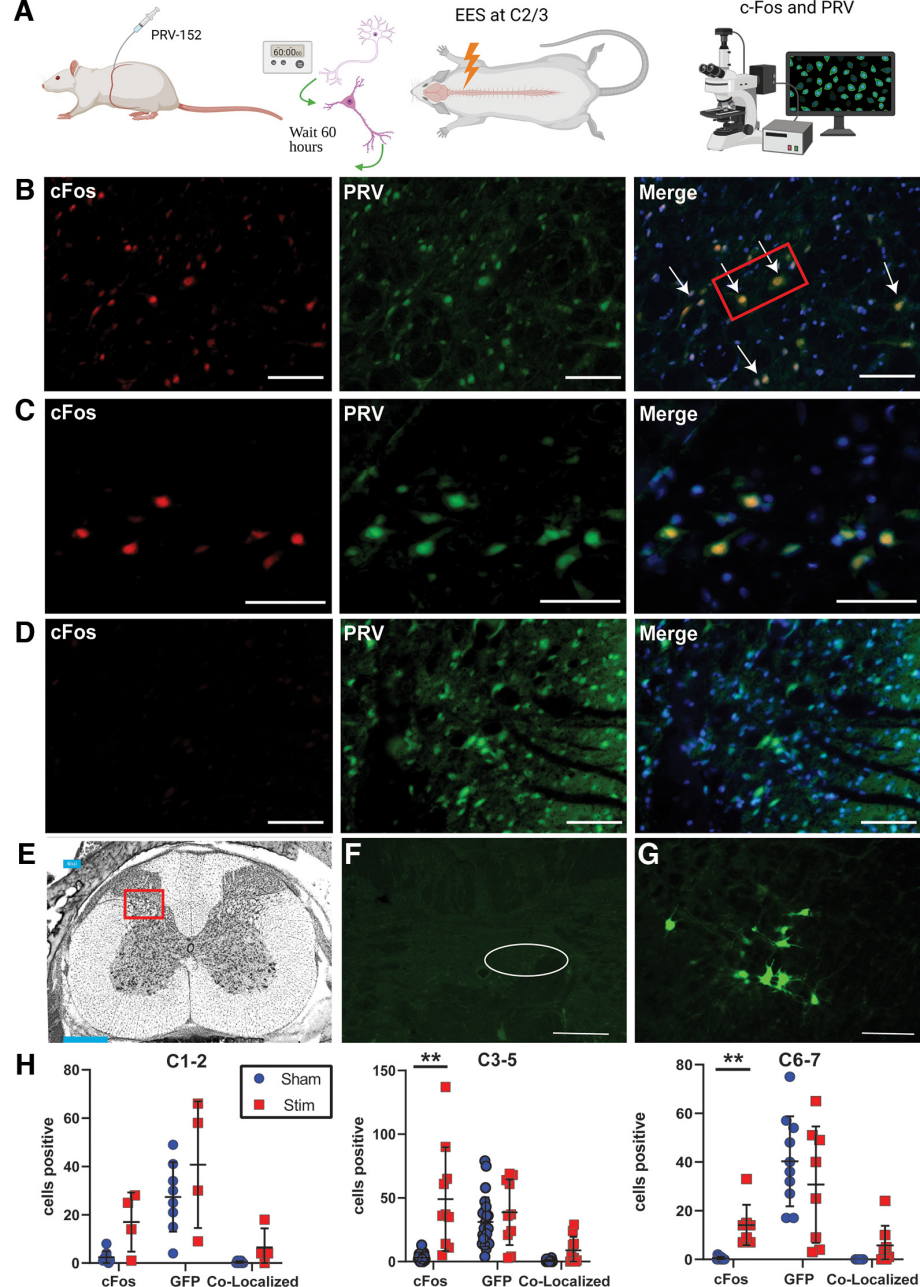
c-Fos expression in respiratory interneurons expressed in animals that received 30-Hz CEES at C2/3. ***A***, Experimental design. ***B***, c-Fos expression observed co-localized with expression of PRV-GFP in the dorsal cervical spinal cord of stimulated animals at C2/3. ***C***, 40× magnification view of the red box region in panel ***B***. ***D***, Little to no c-Fos expression in animals that received sham surgery. ***E***, Graphic showing quantification area highlighted in the red box. ***F***, Dorsal motor nucleus (DMN; circled above) was visualized in animals receiving PRV-152 injections into the diaphragm to ensure that the virus did not leak into the abdominal cavity. The slice shown is ∼−13.8 mm caudal to bregma. Images taken at 4×. Scale bar = 330 μm. ***G***, PRV-152-labeled neurons in the ventrolateral medulla/rostral ventral respiratory group were visualized to ensure premotor neuron infection. The slice shown is at ∼−13.8 mm caudal to bregma. Images taken at 10×. The scale bar = 130 μm. ***H***, c-Fos expression was significantly increased within cervical levels C3–C5 (phrenic nucleus) and C6–C7 compared with animals only receiving PRV-152 retrograde tracing and sham surgery. No differences were observed between groups for expression of PRV-152. There was not a significant difference between groups in the co-localization of c-Fos and PRV-152 expression. All images of ***B*** and ***D*** were acquired at 20× magnification. Scale bar = 70 μm. Images in ***C*** were acquired at 40× magnification. Scale bar = 50 μm. ***A*** was created with BioRender.com. Mean line and error bars represent standard deviation. **p ≤ 0.01. PRV, PRV-152; SST, somatostatin; GFP, green fluorescent protein.

Since CEES modulated respiratory behavior in anesthetized rats, we explored the optimal stimulation location and identified the neurons activated by CEES at C2/3. To investigate spinal neurons connected to the phrenic motor neurons through polysynaptic circuits that may be activated by CEES, the retrograde tracer, pseudorabies virus (PRV-152), was injected bilaterally into the diaphragm muscle. The incubation time used for this experiment (64–66 h) was sufficient to label spinal respiratory-related interneurons, including those labeled before and after brainstem premotor neuron labeling (Dobbins and Feldman, 1994; Lane et al., 2008). For studies of c-Fos activation, a control group of animals received PRV-152 injections into the diaphragm 64–66 h before the laminectomy and received sham treatment; more than an hour was allowed to pass after the laminectomy before euthanizing the animals for tissue extraction. The CEES treatment group received a laminectomy followed by six CEES (Stim) and sham (Sham) trials at C2/3, each separated by a period of 8–10 min to allow respiratory activity to return to baseline (as shown in Fig. 1). Animals in the Stim group were euthanized 1 h after receiving the fourth round of stimulation. For each group, tissue was stained with antibodies against the immediate-early transcription factor protein, c-Fos, and anti-GFP to visualize PRV-152 (Fig. 2). There was minimal to no GFP signal observed in the dorsal motor nucleus, as shown in Figure 2*F*, indicating that the virus did not leak into the abdominal cavity and the staining of neurons in the spinal cord originated from the diaphragm. One animal out of 12 had background and minor staining in the DMN. We included this image to be fully transparent. We compared it to staining in the DMN of PRV-treated animals for tracing afferent projections to the DMN in the literature and felt that the barely detectable leakage from the site of diaphragmatic injection observed across all animals (with one exception) was unlikely to contribute to the staining that we observed in the spinal cord. GFP signal was observed in the ventrolateral medulla, demonstrating that PRV-152, through retrograde infection, entered phrenic motor neurons and moved “upstream” to infect putative spinal interneurons and other rostral elements of the respiratory control system (Dobbins and Feldman, 1994; Fig. 2*G*). Mononuclear infiltration and glia have been observed when PRV-152 is left to replicate for long periods (≥72 h) because of an immune response from the infection (Card et al., 1990; Lane et al., 2008). The control animals showed minimal signs of c-Fos activity, indicating immune cells were not significantly contributing to c-Fos expression. Additionally, c-Fos expression occurred in both PRV-152-labeled and non-PRV-152-labeled neurons in the animals that received active stimulation. Most c-Fos-positive neurons were located in the dorsal horn at each cervical spinal level examined, specifically in laminae 1–3 and to a lesser extent laminae 4–6. c-Fos and PRV-GFP-labeled cells were counted, and the extent of co-localization in laminae 2–5 was assessed, as shown in Figure 2. There was a significant interaction between stain (c-Fos or GFP) and condition (Stim or Sham) within levels C3–C5 (phrenic nucleus- *F*_(2,64)_ = 16.73, df = 2, *p* = 0.0001) and C6–C7 (*F*_(2,32)_ = 4.91, df = 2, *p* = 0.01). There was significantly more c-Fos expression in tissue within the C3–C5 (49.1 ± 40.74 cells, *p* = 0.018) and C6–C7 regions (14.13 ± 8.32 cells, *p* = 0.007), compared with sham animals in comparable regions (C3–C5 3.08 ± 3.05, C6–C7 0.4 ± 0.70), as shown in Figure 2*H*. c-Fos activation was higher at levels closer to the site of the CEES application (C2/3). At all levels, there were more neurons with co-localized c-Fos and GFP expression in the active CEES condition, but this was not significantly different from the sham condition in which each animal breathed normally under anesthesia without CEES (*p* > 0.05). There were no differences in GFP expression between conditions at C1–C2 (Sham 27.38 cells; SD ±14.39 cells, Stim 40.75 cells; SD ±26.20 cells), C3–C5 (Sham 31.13 cells; SD ±19.33 cells, Stim 38.70 cells; SD ±25.67 cells), and C6–C7 (Sham 40.30 cells; SD ±18.50 cells, Stim 30.75 cells; SD ±23.89 cells). These results suggest that CEES activates an expansive network of respiratory-related and nonrespiratory-related cells within the spinal cord, including neurons active in baseline respiratory activity.

**Figure 3.**
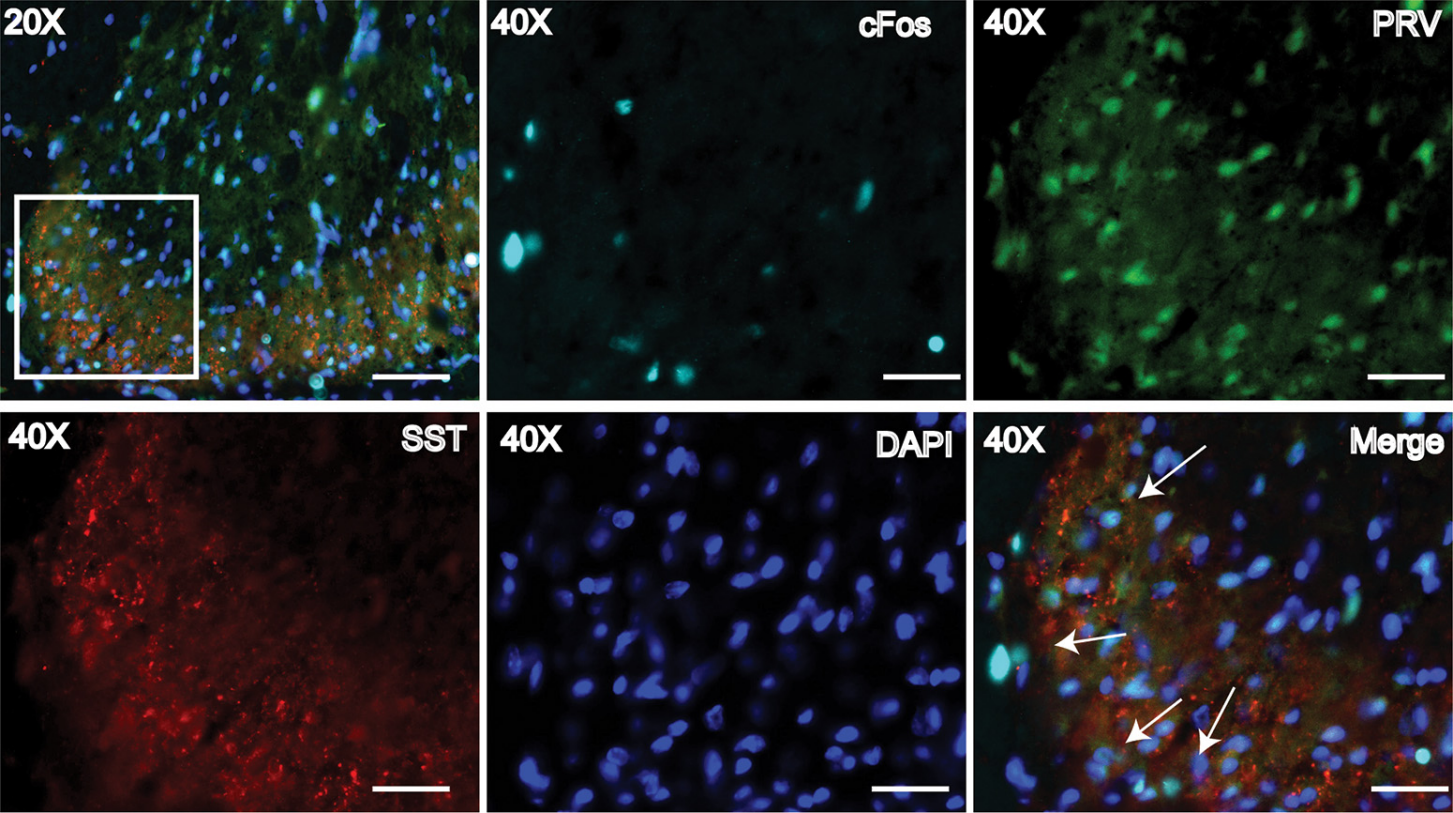
c-Fos-positive respiratory-related interneurons were observed in and around SST expression in the dorsal horn of the cervical spinal cord. Arrows point to c-Fos-PRV-positive neurons near SST expression. 20× scale bar = 70 μm. 40× scale bar = 30 μm. Mean line and error bars represent standard deviation.

### Somatostatin-expressing neurons activated by C2/3 epidural electrical stimulation

Somatostatin (SST) is expressed in neurons within brainstem respiratory nuclei that influence the respiratory phase and timing of neuronal firing within the respiratory pattern generator (Stornetta et al., 2003; Tan et al., 2008; Koizumi et al., 2016). Given this and its known expression in sensory neurons within the spinal cord, we hypothesized that SST expression might also be observed in those respiratory-related neurons in the dorsal cervical spinal cord that also expressed c-Fos after CEES. We found that SST expression was extensive in regions where CEES-induced and respiratory-related c-Fos expression co-localized with putative respiratory interneurons (PRV-152-positive cells; i.e., laminae 1–3, as shown in Fig. 3).

### Epidural electrical stimulation applied along the cervical spinal cord increases ventilation

**Figure 4.**
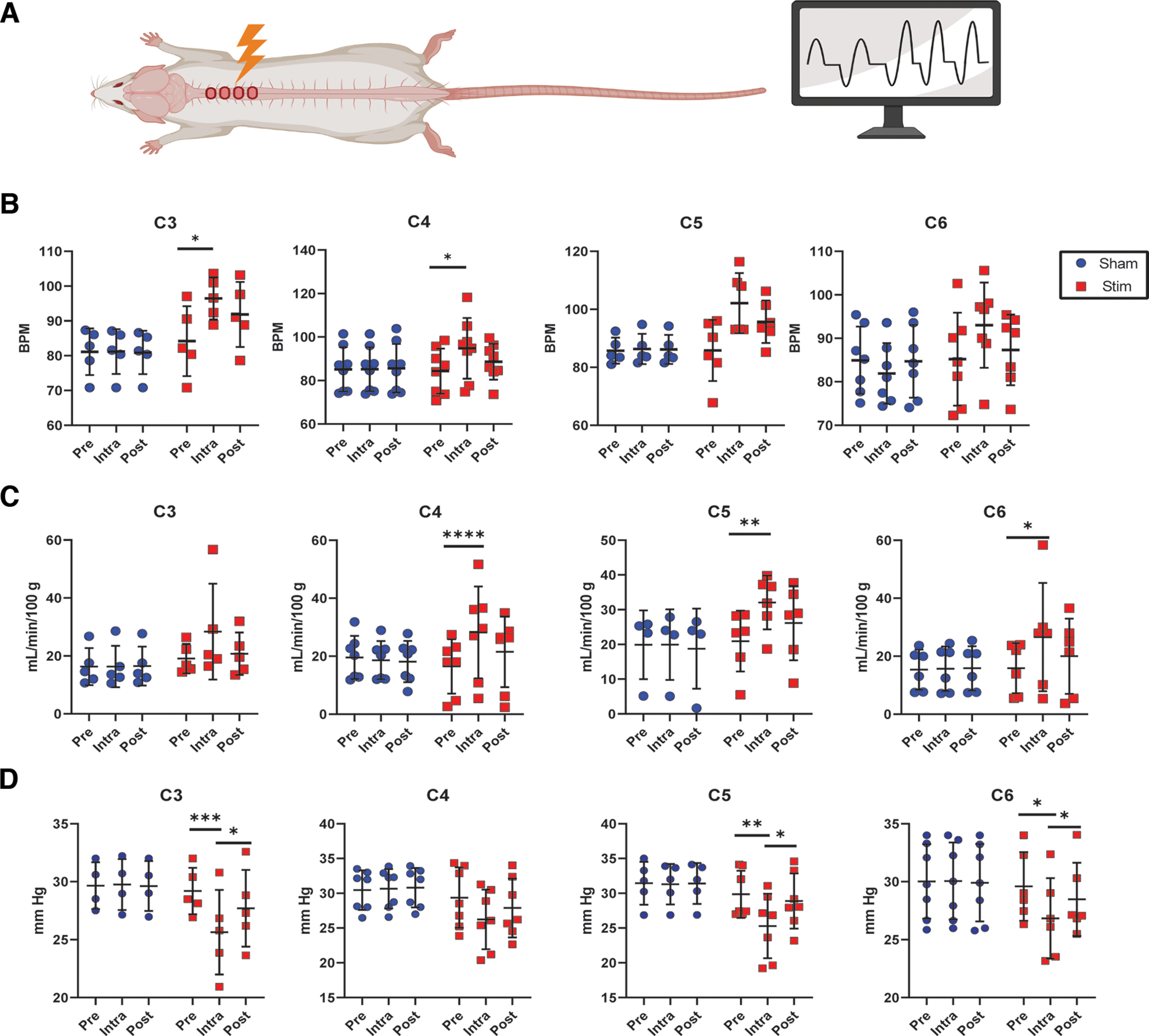
Cervical epidural electrical stimulation at cervical levels 3–6. ***A***, Animals were anesthetized, and CEES was applied while respiratory activity was monitored. ***B***, CEES applied at C3 and C4 increased respiratory frequency compared with baseline frequency. ***C***, CEES applied at C4, C5, and C6 increased minute ventilation compared with baseline. ***D***, Petco_2_ was significantly decreased during CEES when applied at C3, C5, and C6. Data were analyzed with a two-way ANOVA with time (Pre, Intra, Post) and condition (Stim/Sham) for each location. Bonferroni test was applied to account for multiple comparisons. C3 *n* = 5, C4 *n* = 7, C5 *n* = 6, C6 *n* = 6. *p < 0.05, **p < 0.01, ***p < 0.001. Mean line and error bars represent standard deviation. Created with BioRender.com.

Differences in modulation of respiration were previously observed in mice. Therefore, to gain insight into the differences among sites of CEES and respiratory modulation along the cervical spinal cord, where phrenic motor neurons reside, CEES at levels 3–6 (C3–C6) was evaluated (data shown in Table 2; Fig. 4; Huang et al., 2016). There was a significant interaction between time and Stim/Sham for respiratory frequency at C3 (*F*_(2,16)_ = 4.59, df = 2, *p* = 0.02) and C4 (*F*_(228)_ = 4.15, df = 2, *p* = 0.02). Respiratory rate was significantly greater during CEES when applied at C3 (*p* = 0.01) and C4 (*p* = 0.03) compared with baseline (Fig. 4*A*). In addition to frequency modulation, there was a significant interaction between condition and time for minute ventilation when CEES was applied at C4 (*F*_(2,14)_ = 5.01, df = 2, *p* = 0.01). Minute ventilation was significantly greater during CEES at C4 compared with baseline (*p* = 0.001; Fig. 4*B*). CEES applied to C5 and C6 tended to increase frequency, but this effect was not significantly different from sham values. However, there was a significant interaction of condition and time for minute ventilation when CEES was applied at C5 (*F*_(2,10)_ = 6.34, df = 2, *p* = 0.02) and C6 (*F*_(2,10)_ = 4.19, df = 2, *p* = 0.047). When CEES was applied to C5 and C6, there was an increase in minute ventilation compared with baseline (C5 *p* = 0.003, C6 *p* = 0.005; Fig. 4*B*). The interaction between time and condition for tidal volume missed reaching statistical significance (*p* = 0.07). Therefore, frequency-specific effects were observed at more rostral levels (C3 and C4), while the compound increase of frequency and tidal volume increased ventilation at the more caudal levels (C5 and C6). There was a significant interaction between condition and time for Petco_2_ when CEES was applied at C3, C5, and C6. Petco_2_ decreased during stimulation at C3 (*p* = 0.0004), C5 (*p* = 0.007), and C6 (*p* = 0.05) when compared with baseline values, as shown in Figure 4*C*. Petco_2_ during CEES at C4 was lower but not significantly different from baseline (*p* = 0.087). The changes in Petco_2_ reflect changes in alveolar ventilation, which tended to be associated with greater frequency contribution rostrally (C2–C4) and a compound effect of frequency and tidal volume caudally (C5–C6). Although the tidal volume changes were not statistically significant, they were physiologically relevant in that they contributed to increased alveolar ventilation and reduced the Petco_2_ significantly. Sham stimulation had no effect on frequency, minute ventilation, or Petco_2_ (data shown in Table 2), and there was no significant interaction between time, stimulation location, and condition among the four cervical levels tested. Yet, individual two-way ANOVA results suggested differences in the modulation of respiratory activity by CEES along the rostral to caudal axis of the cervical spinal cord, as noted above. In conclusion, CEES increased ventilation in rats and the modulation along the cervical spinal cord varied in terms of frequency or frequency and tidal volume changes depending on the particular location stimulated, similar to observations in anesthetized mice and anesthetized humans (Huang et al., 2016, 2022). Differences in the pattern of respiratory neuromodulation by CEES (enhancing respiratory frequency more or less than tidal volume) along the cervical spinal cord could be exploited therapeutically to enhance different aspects of the respiratory output, favoring tidal volume or respiratory frequency as best suits each patient's needs.

**Table 2.** Mean and SD of the respiratory frequency, minute ventilation, Petco_2_ during sham, and stim trials when CEES was applied at C3, C4, C5, and C6

Variable	Location	Baseline (Pre); mean ± SD	During stimulation (Intra); mean ± SD	Poststimulation (Post); mean ± SD
Condition: CEES				
Respiratory frequency (breaths/min)	C3	84.2 ± 10.0	96.5 ± 6.1*	91.9 ± 9.3
	C4	84.4 ± 10.4	94.8 ± 13.9*	88.6 ± 8.3
	C5	85.8 ± 10.6	102.1 ± 10.4*^n.s.^*	95.7 ± 7.3
	C6	85.2 ± 10.7	93.0 ± 9.8*^n.s.^*	87.3 ± 8.1
Minute ventilation (ml/min/100 g)	C3	19.09 ± 5.01	28.36 ± 16.56*^n.s.^*	20.75 ± 7.34
	C4	16.50 ± 9.36	28.22 ± 15.83****	21.54 ± 12.23
	C5	20.94 ± 8.75	32.08 ± 7.77**	26.16 ± 10.70
	C6	15.86 ± 8.65	26.61 ± 18.68*	20.02 ± 12.97
Petco_2_ (mmHg)	C3	29.19 ± 2.01	25.64 ± 3.66***	27.71 ± 3.31
	C4	29.37 ± 4.34	26.24 ± 4.30 *^n.s.^*	27.89 ± 4.24
	C5	29.86 ± 3.38	25.29 ± 4.64**	28.90 ± 3.96*
	C6	29.59 ± 2.96	26.83 ± 3.48*	28.48 ± 3.16*
Condition: sham				
Respiratory frequency (breaths/min)	C3	81.1 ± 6.7	81.2 ± 6.5	80.9 ± 6.2
	C4	85.2 ± 10.2	85.2 ± 10.1	85.6 ± 11.2
	C5	85.8 ± 4.5	86.3 ± 5.2	86.2 ± 5.0
	C6	84.9 ± 7.7	81.9 ± 6.9	84.7 ± 8.4
Minute ventilation (ml/min/100 g)	C3	16.33 ± 6.40	16.34 ± 7.16	16.49 ± 6.71
	C4	19.57 ± 7.43	18.64 ± 6.59	18.15 ± 7.11
	C5	19.88 ± 9.88	19.93 ± 10.15	18.79 ± 11.54
	C6	15.38 ± 6.96	15.69 ± 7.67	15.86 ± 7.62
Petco_2_ (mmHg)	C3	29.66 ± 2.03	29.76 ± 2.20	29.63 ± 2.16
	C4	30.46 ± 2.84	30.66 ± 2.83	30.81 ± 2.83
	C5	31.44 ± 3.10	31.31 ± 2.92	31.41 ± 2.94
	C6	30.12 ± 3.21	30.06 ± 3.32	29.91 ± 3.35

*p ≤ 0.05,

**p ≤ 0.01,

***p ≤ 0.001,

****p ≤ 0.0001; *n.s.*, not significant.

**Table 3.** Mean and SD of the percent change of respiratory frequency, minute ventilation, and Petco_2_ from baseline

Variable	Group	During CEES, frequency percent change from baseline (% ± SD)	During CEES, minute volume percent change from baseline (% ± SD)	During CEES, Petco_2_ percent change from baseline (% ± SD)
Sham	AAV-SST-Cre+AAV-hM4D(Gi)+CNO	−1.34 ± 2.07	1.67 ± 8.77	0.02 ± 0.33
	AAV-SST-Cre+AAV-hM4D(Gi)+DMSO	−0.88 ± 1.80	−0.26 ± 4.13	0.02 ± 0.41
	AAV-SST-eGFP+AAV-hM4D(Gi)+CNO	−0.17 ± 1.20	0.28 ± 4.05	0.45 ± 0.78
Pre CNO/DMSO Stim	AAV-SST-Cre+AAV-hM4D(Gi)+CNO	31.29 ± 13.80	37.55 ± 26.59	−15.62 ± 4.33**
	AAV-SST-Cre+AAV-hM4D(Gi)+DMSO	24.96 ± 13.84	65.28 ± 39.67	−14.40 ± 4.81***
	AAV-SST-eGFP+AAV-hM4D(Gi)+CNO	22.53 ± 14.60	38.76 ± 19.41	−10.45 ± 6.18**
20 min post-CNO/DMSO	AAV-SST-Cre+AAV-hM4D(Gi)+CNO	26.82 ± 22.09	9.82 ± 48.03	−10.01 ± 7.86*^n.s.^*
	AAV-SST-Cre+AAV-hM4D(Gi)+DMSO	30.03 ± 10.13	78.08 ± 106.78	−20.80 ± 7.75***
	AAV-SST-eGFP+AAV-hM4D(Gi)+CNO	28.52 ± 19.05	42.51 ± 30.63	−10.66 ± 7.87*
40 min post-CNO/DMSO	AAV-SST-Cre+AAV-hM4D(Gi)+CNO	9.10 ± 11.63	15.05 ± 30.32	−6.56 ± 9.33*^n.s.^*
	AAV-SST-Cre+AAV-hM4D(Gi)+DMSO	29.21 ± 15.86	80.05 ± 145.85	−17.03 ± 8.54**
	AAV-SST-eGFP+AAV-hM4D(Gi)+CNO	30.09 ± 14.76	29.80 ± 25.53	−14.20 ± 7.39**
60 min post-CNO/DMSO	AAV-SST-Cre+AAV-hM4D(Gi)+CNO	7.24 ± 12.83	4.93 ± 21.67	−8.04 ± 10.63*^n.s.^*
	AAV-SST-Cre+AAV-hM4D(Gi)+DMSO	23.79 ± 10.48	74.29 ± 146.55	−17.03 ± 7.00**
	AAV-SST-eGFP+AAV-hM4D(Gi)+CNO	34.38 ± 10.07	19.98 ± 24.95	−14.18 ± 10.91*
80 min post-CNO/DMSO	AAV-SST-Cre+AAV-hM4D(Gi)+CNO	19.09 ± 22.39	8.27 ± 26.59	−7.96 ± 7.45*^n.s.^*
	AAV-SST-Cre+AAV-hM4D(Gi)+DMSO	17.85 ± 11.12	63.89 ± 87.47	−16.29 ± 12.07*
	AAV-SST-eGFP+AAV-hM4D(Gi)+CNO	35.53 ± 24.60	30.36 ± 41.97	−15.42 ± 7.12**
100 min post-CNO/DMSO	AAV-SST-Cre+AAV-hM4D(Gi)+CNO	19.55 ± 17.01	5.01 ± 28.60	−7.89 ± 6.29*^n.s.^*
	AAV-SST-Cre+AAV-hM4D(Gi)+DMSO	24.46 ± 9.13	27.62 ± 51.53	−17.10 ± 9.24**
	AAV-SST-eGFP+AAV-hM4D(Gi)+CNO	35.16 ± 17.06	−2.12 ± 32.58	−17.22 ± 6.97***

*p ≤ 0.05,

**p ≤ 0.01,

***p ≤ 0.001; *n.s.*, not significant.

### Silencing SST-expressing neurons decreases the effect of cervical epidural electrical stimulation

**Figure 5.**
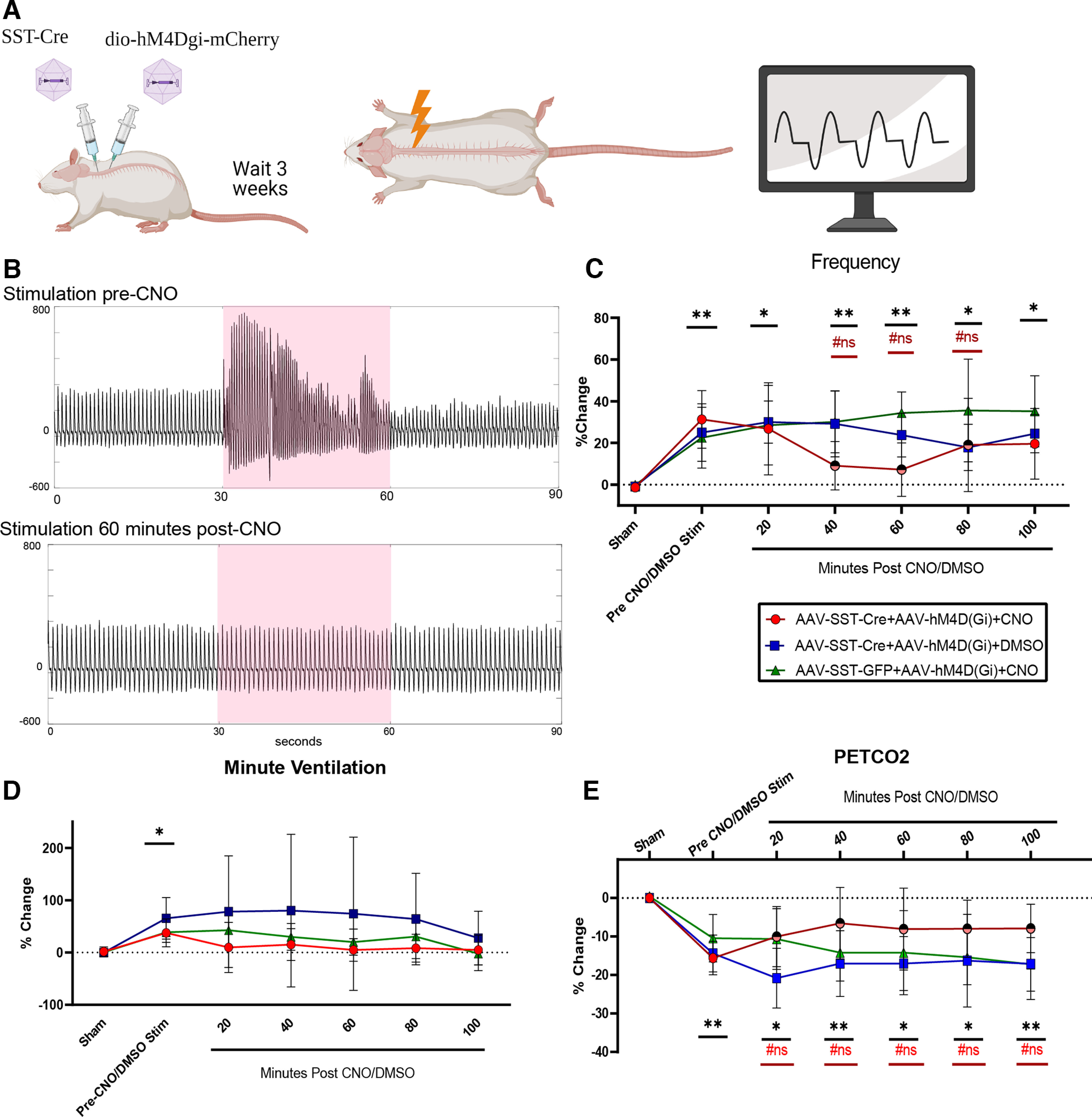
hM4D(Gi) was expressed in SST neurons within the cervical spinal cord and CEES at C3 was conducted before and after a CNO/DMSO (1 mg/kg in 1.5% DMSO) intraperitoneal injection. ***A***, Experimental design. Wild-type rats were injected with dual AAV viral injections to express hM4D(Gi) in SST-expressing neurons. ***B***, Example of raw tracheal pressure from an animal that received 30 s of CEES, in red, before CNO and 60 min post-CNO. ***C***, Mean and SD of respiratory frequency modulation analyzed as a percent change from the baseline respiratory frequency. ***D***, Minute ventilation modulation. ***E***, Mean of Petco_2_ change during CEES across time. Data were analyzed with mixed effects model with group as a between-subjects factor and time as a within-subjects factor. Dunnett's test was used for multiple comparisons. #ns specifies AAV-SST-Cre+AAV-hM4D(Gi)+CNO comparison to Sham stimulation. *n* = 8 per group; *p < 0.05, **p < 0.01. ns, not significant. Mean line and error bars represent standard deviation. Created with BioRender.com

**Figure 6.**
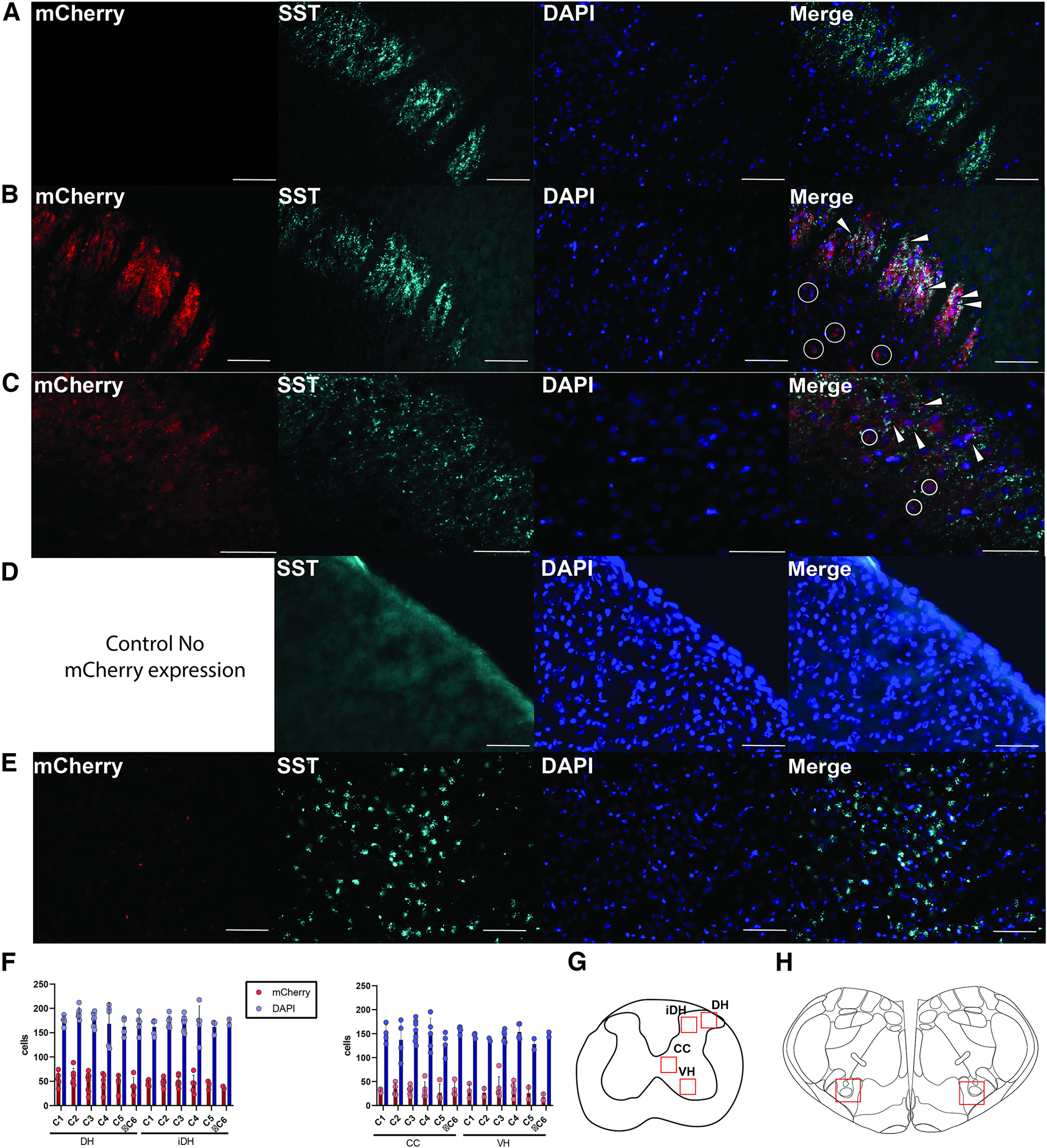
hM4D(Gi) expression verified by mCherry expression. hM4D(Gi) expression was visualized mainly in the dorsal horn. ***A***, hM4DGi-mCherry expression in tissue from an animal that received AAV-SST-eGFP and AAV-dio-hM4D(Gi)-mCherry. ***B***, hM4D(Gi)-mCherry expression at 20× magnification in an animal that received AAV-SST-Cre and AAV-dio-hM4D(Gi)-mCherry. Arrow heads indicate mCherry and SST co-expression. Circles indicate mCherry expression in cells not labeled with the SST antibody. Scale bar = 70 μm. ***C***, hM4D(Gi)-mCherry expression at 40× magnification in an animal that received AAV-SST-Cre and AAV-dio-hM4D(Gi)-mCherry. Arrow heads indicate mCherry and SST co-expression. Circles indicate mCherry expression in cells not labeled with the SST antibody. Scale bar = 30 μm. ***D***, Example of a fluorescence image acquired with tissue that had been incubated with a SST blocking peptide and the primary antibody. ***E***, Minimal mCherry expression was observed in the rostral ventral respiratory group after intraspinal injection of hM4D(Gi) in the cervical spinal cord. Slice shown is at ∼−13.8 mm caudal to bregma at 20× magnification. ***F***, Quantification of mCherry punctate. ***G***, ***H***, Diagrams indicating regions of quantification. Unless specified, all images were acquired at 20× magnification. Scale bar = 70 μm. Mean line and error bars represent standard deviation. SST, somatostatin; DH, dorsal horn; iDH, inner dorsal horn; CC, central canal; VH, ventral horn.

Since SST expression co-localized with CEES-induced c-Fos expression, and SST-positive neurons may participate in respiratory activation during CEES, we hypothesized that inhibiting SST-expressing neurons might reduce the respiratory response to CEES. To test this hypothesis, we selectively expressed the chemogenetic inhibitory Designer Receptor Exclusively Activated by Designer Drugs (DREADD), hM4D(Gi), in SST-expressing cells in the cervical spinal cord (Fig. 5*A*). Selective expression of hM4D(Gi) in SST cells was probed and verified by co-localization of mCherry expression and SST expression in the dorsal horn of the cervical spinal cord and minimal mCherry expression that was not associated with SST immunofluorescence (Fig. 6). A low dose of CNO (1 mg/kg) was used to silence SST-expressing cells that also expressed hM4D(Gi) (MacLaren et al., 2016). We studied the following groups of animals: AAV-SST-Cre+AAV-hM4D(Gi)+CNO, AAV-SST-Cre+AAV-hM4D(Gi)+DMSO, AAV-SST-eGFP+AAV-hM4D(Gi)+CNO. There was a significant interaction between group and time for respiratory frequency (*F*_(12,123)_ = 2.78, *p* = 0.002). All groups responded with a significant increase in respiratory frequency during CEES at C3 before drug delivery compared with sham trials (AAV-SST-Cre+AAV-hM4D(Gi)+CNO *p* = 0.001, AAV-SST-Cre+AAV-hM4D(Gi)+DMSO *p* = 0.005, AAV-SST-eGFP+AAV-hM4D(Gi)+CNO *p* = 0.01; Table 3, Fig. 5). Respiratory frequency increased significantly during CEES at all time points tested (20–100 min post-DMSO/CNO delivery) in rats that were in either control group (AAV-SST-Cre+AAV-hM4D(Gi)+DMSO or AAV-SST-GFP+AAV-hM4D(Gi)+CNO). In contrast, when CEES was applied in the active (inhibitory) experimental group (AAV-SST-Cre+AAV-hM4D(Gi)+CNO), the increase in respiratory frequency was diminished or ablated at 40, 60, and 80 min after CNO delivery, as shown in Table 3 and Figure 5*B*,*C*. This reduction was sufficient to return the frequency to values equal to those observed in the sham group (40, *p* = 0.16, 60, *p* = 0.41, 80, *p* = 0.18). Volumes of AAV vectors ranged from 100–700 nl per injection. Larger injection volumes were associated with greater inhibition of the response to CEES and injection volumes ≥400 nl caused maximal inhibition, resulting in close to zero change from baseline during stimulation. There was no significant interaction between time and group for minute ventilation responses among groups over time, similar to what was seen during stimulation at C3 in the prior experiment (Fig. 5*D*). We found a significant interaction between group and time for changes in Petco_2_ (*F*_(12,108)_ = 1.92, df = 12, *p* = 0.039), indicating that alveolar ventilation increased significantly during CEES. Before CNO/DMSO treatment, Petco_2_ decreased in all treatment groups (AAV-SST-Cre+AAV-hM4D(Gi)+CNO *p* = 0.004, AAV-SST-Cre+AAV-hM4D(Gi)+DMSO *p* = 0.0003, AAV-SST-eGFP+AAV-hM4D(Gi)+CNO *p* = 0.004; Table 3 and Fig. 5*E*). After CNO treatment, reductions in Petco_2_ were not significantly different from sham in the active, inhibitory, experimental group (AAV-SST-Cre+AAV-hM4D(Gi)+CNO). The Petco_2_ decreased in the remaining control groups when CEES was applied throughout the 100 min of the experiment. These results suggest that, close to the site of stimulation at C3, the respiratory frequency modulation induced by CEES was dependent on neurons expressing SST.

Expression of mCherry was quantified across cervical levels to determine where hM4D(Gi) was expressed. The majority of mCherry was expressed within the dorsal horn, as shown in Figure 6. Rostral brainstem slices were examined for mCherry expression to determine the extent of the brainstem contribution to hM4D(Gi)-mediated inhibition during CEES modulation of respiration. Scant mCherry expression was observed within the brainstem, as shown in Figure 6*E*. This suggests that the majority of hM4D(Gi) was expressed in the cervical spinal cord, diminishing the likelihood that DREADD-induced inhibition of brainstem respiratory neurons contributed to the respiratory inhibition when hM4D(Gi) was activated by CNO. Thus, the hM4D(Gi) acted by inhibiting the activity of SST-expressing dorsal spinal neurons (likely interneurons) to occlude the excitatory effect of CEES on respiration.

## Discussion

Respiratory activity increased during CEES applied to the dorsal spinal cord of anesthetized rats. Using c-Fos as a neuronal activation marker, we found that CEES activated spinal neurons mainly in the dorsal horn. To characterize presumed respiratory-related neurons, PRV-152 was injected into the diaphragm, and c-Fos was co-localized with PRV-152-labeled putative interneurons as well as non-PRV-152-labeled neurons. There was no difference in co-localization of c-Fos and PRV-152 between sham and stimulated animals suggesting that a basal activation state existed in the unstimulated condition. CEES may have enhanced the already active spinal respiratory network plus additional respiratory and nonrespiratory spinal neurons. c-Fos expression is not sensitive to measure the degree of activation, only the presence or absence of activation. Since somatostatin (SST) is expressed in neurons in the brainstem expressing respiratory-related activity, we tested the hypothesis that SST-expressing neurons in the cervical spinal cord participate in the respiratory activation mediated by dorsal CEES (Tan et al., 2008; Smith et al., 2013). Co-localization of SST, c-Fos, and PRV-152 was observed in the dorsal horn, indicating that SST-expressing neurons could be mediating respiratory responses elicited by CEES. Finally, using dual-viral vector injections of Cre downstream of the SST-promoter and an hM4D(Gi) double-floxed vector, we selectively expressed the inhibitory DREADD, hM4D(Gi), in spinal SST-expressing neurons. Inhibition of the spinal SST-expressing neurons suppressed CEES-induced increases in respiratory frequency and alveolar ventilation and decreases in Petco_2_. The increase in minute ventilation elicited by CEES was also reduced following treatment with CNO, but minute ventilation responses tended to be more variable across all groups and no significant differences were seen. Our results suggest that CEES activates SST-expressing neurons within a sensory, respiratory-related, neuronal network in the cervical spinal cord that can enhance respiratory activity. Some caution is warranted when interpreting the magnitude of the effect of inhibiting SST-expressing neurons; the arithmetic of inhibiting different types of cells does not always sum to one. Although inhibition of SST-expressing neurons completely blocked the effect of CEES in these studies, there may still be other non-SST expressing cell types involved in this process, especially if we were to observe the effect of inhibition of SST-expressing neurons over a longer time or in awake animals.

Phrenic nerve stimulation, ventral or dorsal epidural stimulation along the cervical and thoracic spinal cord, and intraspinal stimulation using bursts of stimulation (usually high frequency, >100 Hz) to pace diaphragmatic muscle activity enhanced respiratory activity (Kowalski et al., 2013; Le Pimpec-Barthes et al., 2016; Mercier et al., 2017; Reynolds et al., 2017; Bezdudnaya et al., 2018; Sunshine et al., 2021). In contrast, we explored the effects of low-intensity, continuous (unpaced) dorsal CEES on respiration. The use of continuous 30-Hz stimulation was based on the beneficial effects of similar stimulation activating rhythmic spinal neural networks (Dimitrijevic et al., 1998; Lavrov et al., 2008; Huang et al., 2016, 2022). The amplitude of stimulation needed to excite respiratory activity in our study depended on anesthesia depth, animal size, and tissue resistance. In some animals, higher intensity stimulation inhibited respiratory activity and resulted in periods of apnea (D. C. Lu, unpublished observation), likely because of direct motor neuron activation resulting in tetanic contraction since higher intensity caused tetanic contraction of the proximal upper limb muscles. We selected a stimulation amplitude that minimized extra-respiratory muscle contraction and maximized respiratory modulation. Thus, continuous, low intensity, low-frequency neuromodulation below the threshold of direct motor activation enhanced respiratory activity during CEES without directly pacing respiratory motor neurons, as previously shown in anesthetized mice and anesthetized humans (Huang et al., 2016, 2022).

Given the incubation time (64 h) of PRV-152, which allowed multiple synaptic jumps, PRV-152 likely labeled interneurons that receive projections from diaphragmatic afferents (Lane et al., 2008). Dorsal interneurons expressing SST are excitatory and receive input from Type II, III, and IV fibers that transmit mechano-sensation and nociceptive information (Proudlock et al., 1993; Xu et al., 2013; Duan et al., 2014; Gutierrez-Mecinas et al., 2016; Chamessian et al., 2018). The phrenic nerve has numerous types of afferents, Iα, Iβ, II, III (Iδ), and IV, that influence respiratory activity (Marlot et al., 1987; Bałkowiec et al., 1995; Nair et al., 2017b). Since c-Fos immunofluorescence was observed in the dorsal horn, CEES likely modulates respiratory activity through a sensory circuit involving SST-expressing interneurons in layers I–IV of the dorsal horn, possibly by activating Type III and IV phrenic nerve afferents (Marlot et al., 1987; Ward et al., 1992; Yu and Younes, 1999; Malakhova and Davenport, 2001; Nair et al., 2017b). Phrenic afferents project to spinal interneurons in laminae 1–4, 7, and 10 in close proximity to the site of c-Fos and PRV-152 co-localization (Goshgarian and Roubal, 1986; Nair et al., 2017a, b). EES at lumbar levels preferentially activates sensory neurons leading to monosynaptic and polysynaptic interneuronal activation of motor neurons (Struijk et al., 1993; Holsheimer, 2002; Capogrosso et al., 2013), and activation of similar polysynaptic circuits in the cervical spinal cord may enhance diaphragm activity.

Inhibition of SST-expressing neurons in the cervical spinal cord diminished CEES-induced respiratory activation (Fig. 5). There are two possible mechanisms for the effects of CEES, an intraspinal pathway and a supraspinal pathway. Activating cervical SST-expressing interneuron likely enhances an excitatory polysynaptic sensory circuit within the spinal cord, which then communicates with and enhances phrenic motor neuron excitability when phrenic motor neurons are activated by descending central pattern generator activity. Consistent with such a hypothesis, tonic diaphragmatic activity increased between inspiratory phases. The modulation of respiratory activity by CEES-elicited afferent activity is likely derived from spinal and supraspinal actions. Laminae 2–4, areas in which we found higher c-Fos-positive cells in animals receiving stimulation, transmit information from primary sensory afferents to higher brain structures in addition to local spinal interneurons (Fig. 3; Willis et al., 2001; Todd, 2010; Braz et al., 2014). Neurons in lamina 3 provide polysynaptically projections to neurons in lamina 1 where projections ascend to higher brain structures (Baba et al., 2003; Lu et al., 2013). CEES is likely activating sensory fibers traveling via the fasciculus cuneatus, sending information to the medulla, thalamus, and primary somatosensory cortex, all of which can influence respiratory activity (Bolser et al., 1991; Malakhova and Davenport, 2001; Zhang and Davenport, 2003; Davenport et al., 2010). From above the spinal cord, the pons and medulla provide descending propriospinal premotor connections to phrenic motor neurons in the cervical spinal cord and cranial and more caudal spinal motor neurons that innervate respiratory muscles (Koshiya et al., 2014; Huckstepp et al., 2015; Ikeda et al., 2017). Given minimal hM4D(Gi) expression observed in the brainstem, the diminished respiratory modulation during CEES after inhibiting SST-expressing neurons in the spinal cord likely originates locally within the spinal cord (Fig. 6). Activation of this spinal sensory circuit by CEES is likely to increase the activity of the ponto-medullary circuit by ascending CEES-induced excitation of sensory spinal pathways so that in addition to sensitizing spinal motor neurons to descending inputs, CEES may increase the overall drive by increasing excitatory inputs to phrenic motor neurons and other respiratory motor neurons.

SST-expressing neurons in the spinal cord are heterogenous, and SST neurons in deeper lamina express inhibitory neurotransmitters (Proudlock et al., 1993). An inhibitory circuit could also explain the results that we found, but would require inhibition of inhibitory respiratory inputs so that facilitation of respiratory activity could emerge. Inhibitory influences on phrenic nerve output both from supraspinal and intraspinal processes may originate from activation of Renshaw cells and could serve this function (Hilaire et al., 1986; Parkis et al., 1999; Marchenko and Rogers, 2009; Zaki Ghali et al., 2019).

We conclude that dorsal CEES activates a spinal circuit that enhances respiratory activity. All cervical stimulation sites modulated respiratory activity; however, the respiratory frequency increased after rostral stimulation, whereas minute ventilation increased significantly, with less frequency modulation, after caudal stimulation. Differences in neuromodulatory responses to CEES along the cervical spinal cord could provide a tool to tailor respiratory output toward increased frequency or tidal volume to personalize the respiratory enhancement to an individual's specific deficit. Somatostatin-expressing neurons appear to mediate some part of the spinal response to CEES that is, in turn, communicated to the respiratory control system and respiratory motor neurons. Future work to dissect this respiratory sensory-motor circuit should explore how the spinal circuit activated by CEES interacts with brainstem and spinal motor nuclei so that the findings in our proof-of-principle study maybe transformed into a therapeutically useful tool. Additional preclinical experiments in animal models may provide a framework for CEES and its use to maintain spontaneous respiratory activity in cases of diminished respiratory activity where it may be possible to combine pharmacological or genetic interventions directed at SST-expressing neurons to maintain or augment specific patterns of respiratory activity.
